# Prevalence of molecular subtypes and prognosis of invasive breast cancer in north-east of Morocco: retrospective study

**DOI:** 10.1186/1756-0500-5-436

**Published:** 2012-08-13

**Authors:** Sanae Bennis, Fouad Abbass, Yousra Akasbi, Kaoutar Znati, Khalid Amrani Joutei, Omar El Mesbahi, Afaf Amarti

**Affiliations:** 1 Department of Pathology, Laboratory Biology of cancers-Faculty of Medicine & Pharmacy, Hassan II University Hospital Fez, Km 2.200 Route de Sidi Harazem, Fez, Morocco; 2 Faculty of Sciences and Technology, Fez, Morocco; 3 Department of Pathology, Hassan II University Hospital, Fez, Morocco; 4 Medical Oncology unit, Hassan II University Hospital, Fez, Morocco

**Keywords:** Breast cancer, Molecular subtypes, Prognosis, Overall survival (OS), Disease free Survival (DFS)

## Abstract

**Background:**

Breast carcinoma is known as a heterogeneous disease because gene expression analyses identify several subtypes and the molecular profiles are prognostic and predictive for patients. Our aim, in this study, is to estimate the prevalence of breast cancer subtypes and to determine the relationship between clinico-pathological characteristics, overall survival (OS) and disease free survival (DFS) for patients coming from north-east of Morocco.

**Methods:**

We reviewed 366 cases of breast cancer diagnosed between January 2007 to June 2010 at the Department of pathology. Age, size tumor, metastatic profile, node involvement profile, OS and DFS were analyzed on 181 patients. These last parameters were estimated by Kaplan-Meier analysis and log-rank test to estimate outcome differences among subgroups.

**Results:**

The average age was 45 years, our patients were diagnosed late (57% stage III, 17.5% stage IV) with a high average tumor size. Luminal A subtype was more prevalent (53.6%) associated with favorable clinic-pathological characteristics, followed by luminal B (16.4%), Her2-overexpressing (12.6%), basal-like (12.6%) and unclassified subtype (4.9%).

Survival analysis showed a significant difference between subtypes. The triple negative tumors were associated with poor prognosis (49% OS, 39% DFS), whereas the luminal A were associated with a better prognosis (88% OS, 59% DFS). The luminal B and the Her2-overexpressing subtypes were associated with an intermediate prognosis (77% and 75% OS, and 41% and 38% DFS respectively).

**Conclusion:**

This study showed that molecular classification by immunohistochemistry was necessary for therapeutic decision and prognosis of breast carcinoma. The luminal A subtype was associated with favorable biological characteristics and a better prognosis than triple negative tumors that were associated with a poor prognosis and unfavorable clinic-pathological characteristics.

## Background

Breast cancer is the most common malignancy in women and the leading cause of cancer mortality in worldwide, it is responsible of more than 500 000 deaths annually [1]. In Morocco, it’s the first cancer in women and is currently a major public health problem. This cancer is a heterogeneous disease encompassing a wide range of clinical behaviors, even in patient groups which appear to be clinically similar. The pathological and clinical heterogeneity, partially responsible for therapeutic failures and reflects its complex molecular basis.

The molecular classification in breast carcinomas is now based upon gene expression analysis using DNA microarrays and allows to identify at least five groups: luminal A, luminal B, HER2-overexpressing, basal-like and normal breast-like [2-4]. However, large-scale subtyping using gene expression profiling from formalin-fixed, paraffin-embedded samples is not currently feasible and remains very expensive. Therefore, immunohistochemical markers have been used as surrogates tools for DNA microarray in subtyping breast cancer [5,6]. Several studies used routinely panels of immunohistochemical markers to classify breast cancers into subtypes similar to those previously defined using gene expression analyses [7]. The most interesting study was realized by *Carey* et al. [5]. They defined several immunohistochemical subtype : luminal A (ER positive (ER+) and/or PR positive (PR+), Her2 negative (Her2-)), luminal B (ER + and/or PR+, Her2 positive (Her2+), Her2+/ER − subtype (Her2+, ER−, PR−) and basal-like (ER−, PR−, Her2−, cytokeratin 5/6 positive (CK5/6+) and/or Her1+ (EGFR)). Tumors which were negative at immunohistochemical staining for all markers (ER, PR, Her2, Her1, and CK5/6) were considered unclassified subtype [5]. According to this classification, we performed immunohistochemical staining for ER, PR, Her2, Her1, CK8/18, basal CK5/6 and CK14 in paraffin sections from blocks of breast cancer. The aim of the present study was to estimate the prevalence of breast cancer subtypes in patients the north east region of Morocco, and to correlate between clinical and pathological characteristics with survival (disease-free survival (DFS) and overall survival (OS)).

## Methods

### Study patients

Four hundred and thirty patients were diagnosed with breast cancer during the period of January 2007 to June 2010, among these women 64 cases were excluded because of lack of one clinicopathological factor such as age or tumor size. Therefore only 366 cases were included in this study. Most of the patients were referred to us either from regional hospitals or gynecology department of Hassan II University Hospital and rarely from other departments. Very few patients were referred from private hospitals. All breast lesions classified by mammography as score 4 or 5 according to ACR classification were confirmed by biopsy using core needle or tru-cut biopsy. In addition, the age of the patient, tumor size, lymph node and metastatic profiles were determined in the Department of Pathology. OS and DFS were analysed on 181 patients and these data were obtained from the Medical Oncology Unit. This study was approved by the ethics committees of both Hassan II University Hospital and Faculty of Medicine and Pharmacy of Fez.

### Histological evaluation

Tumor size was measured in the freshly resected specimens. Tumor samples were subsequently fixed in neutral buffered formaldehyde and processed to paraffin blocks according to standard procedures. Four micrometer thick sections were cut and stained with hematoxylin and eosin for histopathology. The histological classification was based on the criteria set by the World Health Organization (WHO). The histological grade is based on the Scarff-Bloom-Richardson grading system (SBR).

### Immunohistochemical study

Tumors sections were deparaffinized and rehydrated. Peroxide blocking was done with 0.4% H_2_O_2_. Antigen retrieval was achieved by heat retrieval using a pressure cooker. After washing, the slides were treated with protein blocking agent (UltraTech HRP, Immunotech) then incubated with the following primary antibodies: anti-human ER (ER1D5, Immunotech), PR (PR10A9, Immunotech), CK5/6 (D5/16B4, Cell Marque), CK14 (LL002, Cell Marque), CK8/18 (RTU-5D3, Novocastra) and Her1 (EGFR) (3C6, Vantana). After rinsing with PBS, the slides were incubated with a secondary biotinylated antibody (Immunotech). The slides were then rinsed with PBS. Sections were then incubated with streptavidin-peroxidase reagent. Staining for the slides was developed with Amino-Ethyl-Carbazole (Ultra Tech AEC, Immunotech) and then the slides were counterstained with hematoxylin, hydrated, and mounted.

For Her2, immunohistochemical was carried out using with HercepTest (A0485, Dako) according to the commercial instructions for use.

### Immunohistochemical surrogate biomarkers of molecular classification

According to *Carey* et al. [5], immunohistochemical subtypes were defined as follows: luminal A (ER + and/or PR+, Her2-), luminal B (ER + and/or PR+, Her2+), basal-like (ER-, PR-, Her2-, and CK5/6+, Her1+ and/or CK14+), Her2+/ER-, and unclassified subtype (negative for all markers) (Table1). CK8/18 expression was used for confirmation the luminal subtypes. 

**Table 1:** Immunohistochemical characterization of molecular subtypes of breast cancer

**Molecular subtypes**	**Immunohistochemical characterization**
Luminal A	ER + and/or PR+, HER2- CK8/18+
Luminal B	ER + and/or PR+, HER2+, CK8/18+
HER2+	ER-, PR-, HER2+
basal-like	ER-, PR-, HER2- and CK 5/6+, HER1+ and/or CK14+
Unclassified	ER-, PR-, HER2-, CK 5/6-, HER1- and CK14-

ER = estrogen receptor; PR = progesterone receptor; HER2 = human epidermal growth factor receptor 2; HER1 = human epidermal growth factor receptor 1; CK = cytokeratin.

### Fluorescence in situ hybridization (FISH) study

All Her2 score 2+ cases were analyzed by FISH. They were performed using the PathVysion HER2 DNA Probe (Abbott Vysis Inc., Downers Grove, IL) according to the manufacturer’s protocol. The probe cocktail included the LSI HER-2/neu probe and the CEP17 probe. Fluorescence signals were analyzed and digitalized using the CytoVision^TM^ image analysis system (Applied Imaging International Ltd., Newcastle-Upon-Tyne, UK). Between 60 and 100 nuclei were scored for each case. Signal ratios (HER2: CEP17) ≥ 2 were classified as amplified. In the absence of positive FISH data, tumors scored 2+ by IHC were considered as negative for HER-2.

### Quality control and scoring

Positive controls were included in each staining run and consisted of breast cancers known to express each of the antigens of interest. Staining results were evaluated by at least two pathologists. Cases were considered positive for ER and PR according to standardized guidelines using a cut-off of ≥10% stained tumour nuclei by Group for Evaluation of Prognostic Factors using Immunohistochemistry in Breast Cancer (GEFPICS-FNCLCC). Similarly, staining with CK5/6, CK14, CK8/18 and Her1 antibodies were considered as positive when more than 10% of the tumor cells were labeled.

Her2 was scored based on a 0 to 3 scale according to the criteria set by ASCO (American Society of Clinical Oncology/College of American Pathologists) [8]. Scores 0 and 1+ were considered as negative; score 2+ was considered borderline; and score 3+ was considered as strongly positive. FISH was performed on the borderline cases (score 2+).

### Treatment modalities

Treatment modalities were surgery (radical mastectomy or conservative surgery), standard neoadjuvant chemotherapy, adjuvant chemotherapy (anthracycline and taxanes – based regimen), adjuvant trastuzumab and hormonal therapy (tamoxifen).

### Statistical analysis

Statistical analysis was performed in the Department of Epidemiology, of the Faculty of Medicine and Pharmacy of Fez and was carried out using Epi-Info™ 3.4.Version.

Patients were subdivided into groups based on the definition of breast cancer subtypes. Differences between breast cancer subtypes with regard to clinicopathological characteristics were examined using analysis of variance, chi-square tests or Fisher’s exact test. Overall survival (OS) was determined as the length of time from the date of surgery until either the date of death (from any cause) or the date of last follow-up. Disease free survival (DFS) was defined as the time elapsed from the date of surgery to any relapse or death. OS and DFS rates were estimated by Kaplan-Meier analysis and a log-rank test was used to estimate outcome differences among subgroups.

Cox’s regression model was also used to examine several combinations and interactions of different prognosis factors in multivariate analysis and results are presented with 95% confidence intervals (95% CI). A value of p ≤ 0.05 was considered as statistically significant.

## Results

The study was achieved on 366 patients diagnosed with infiltrating breast cancer and managed at the Medical Oncology Unit in Hassan II University Hospital in Fez. Mammography was carried out for all patients according to ACR classification; 8% of lesions were ACR3, 42% were ACR4 and 50% were ACR5. However, 70% of all patients showed a suspect image among which 80% had malignant cells with cytological examination. The patient’s average age at diagnosis was 46.8 ± 12 years (ranging from 18 to 82 years). Seventeen percent (17%) were aged below 35 years old. The tumor clinical stage on first diagnosis, according to American Joint Committee on Cancer Staging Systems, showed that 47 women (14.5%) are at stage I, 108 (33%) are at stage II, 114 (35%) are at stage III and 57 (17.5%) at stage IV. The tumor clinical stage for 40 women was not determined.

After histological analysis, the tumor average size was 3.7 ± 2.6 cm (ranging from 0.2 to 16 cm). Most of these tumors (87,4%) were diagnosed as invasive ductal carcinoma while 4% were invasive lobular carcinomas, 3% were metaplastic carcinoma, 2% were medullary carcinoma and few patients had cancers of rare histology (3%), which were summarized as “other types” in our study. The clinicopathological and histological parameters were presented in Table2.

**Table 2:** Description of characteristics of the study population

**Characteristics**	**Number of cases (%)**	
**Mean age ± SD, median (years)**	**46.8 ± 12, median 45**	
Age (years)		
≤ 35	62	17%
> 35	304	83%
Histological grade SBR		
I	53	15%
II	200	55%
III	111	30%
Average tumor size ± SD (cm)	3,7+/−2,6	
Tumor size (cm)		
≤ 2	100	27%
> 2	266	73%
Histologic type		
Invasive ductal carcinoma	320	87.4%
Invasive lobular carcinoma	15	4%
Medullar carcinoma	7	2%
Metaplasic carcinoma	11	3%
Other types	11	3%
AJCC stage		
I	47	14,5%
II	108	33%
III	114	35%
IV	57	17.5%
Lymph node status		
N0	115	35.4%
N+	211	64.6%
Metastasis status		
M0	269	82.5%
M1	57	17.5%
vascular emboli positive	131	35.8%
HR positive	259	70.8%
ER positive	204	55.7%
PR positive	236	64.5%
Her2 positive	106	29%

The histological grade distribution were grade II (54.7%), grade III (30.7%) but few patients only were grade I (14,6%). Vascular emboli were detected in 35.8% of our patients. The status of lymph nodes and distant metastasis was determined for 326 patients among which 53% had positive lymph nodes and 17.5% had distant metastasis.

The immunohistochemical study showed that 259 patients (70.8%) were ER or/and PR positives, 204 (55.7%) were ER positive, 236 (64.5%) were PR positive, and 106 (29%) were Her2 positive. Therefore, 196 tumors (53.6%) were classified as luminal A, 60 (16.4%) as luminal B, 46 (12.6%) as Her2-overexpressing, 46 (12.6%) as basal-like, and 18 (4.9%) as unclassified subtype (Table3).

**Table 3:** Prevalence of intrinsic subtypes and clinico-pathological characteristics

**Characteristics**	**Luminal A**	**Luminal B**	**Her2+**	**Basal like**	**Unclassified**	*p-*value*
No. Of cases (%)	196 53,6%	60 16,4%	46 12,6%	46 12,6%	13 4,9%	
Mean age (years)	47 ± 12	46 ± 12	45 ± 8	49 ± 14	44 ± 14	0.4628
Mean tumor size (cm)	3.6 ± 2,5	3.9 ± 2,2	3.4 ± 2	4.4 ± 3	3,9 ± 4,3	0.5673
Age groups (years)						
≤ 35	35 17.8%	9 15%	8 17.4%	5 11%	5 28%	0.5074
> 35	161 82.2%	51 85%	38 82.6%	41 89%	13 72%	
Tumor size (cm)						
≤ 2	54 27.6%	16 26,7%	14 30%	9 20%	7 39%	0.8620
> 2	142 72.4%	44 73,3%	32 70%	37 80%	11 61%	
Histological grade (%)						
I	37 18.8%	8 13.3%	6 13%	3 6,6%	0 0%	0.0053
II	114 58.2%	35 58.3%	21 45.6%	21 45.6%	8 45%	
III	45 23%	17 28.3%	19 41.4%	22 47.8%	10 55%	
Vascular emboli						
Negative	124 63.3%	36 60%	28 61%	33 71.7%	14 78%	0.5028
Positive	72 36.7%	24 40%	18 39%	13 28.3%	4 22%	
Lymph node status						
N0	66 36%	14 26%	14 35%	15 45%	6 37,5%	0.5555
N+	117 64%	40 74%	26 65%	18 55%	10 62,5%	
Metastasis						
M0	151 85%	39 76%	34 87%	35 81%	15 67%	0.4055
M+	27 15%	12 24%	5 13%	8 19%	5 33%	

* Fisher test or Chi-square test.

The basal-like subtypes present a higher median tumor size than others subtypes (4.4 cm), followed by unclassified subtype, luminal B (3.9 cm) and Her2-overexpressing (3.4 cm) respectively. In addition, the basal-like subtype had the highest percentage of tumors, exceeding 2 cm (80%).

IHC subtypes were significantly different by histological grade (p = 0.0053). The unclassified, basal-like and Her2-overexpressing subtypes represented a higher percentage of cases with histological grade III (53%; 47.6% and 42.2% respectively), and a very low percentage of tumors with histological grade I (0%, 4.8% and 13.3%, respectively).

The unclassified and basal-like subtypes had less vascular emboli than other subtypes (22% and 28.3% respectively). Luminal B and Her2-overexpressing subtypes had the highest percentage of vascular emboli (40% and 39% respectively).

In this study, the luminal B tumors had the highest percentage of lymph node metastasis (74%) while the basal-like subtype had the lowest percentage (55.2%). The unclassified and luminal B subtypes patients had a higher percentage of distant metastasis than other subtypes (33% and 24% respectively).

Our study evaluated complete pathologic response with standardized neoadjuvant therapy (taxan or anracycline) in 26% of patients and showed that complete pathologic response rates was found in 17% of patients (62.5% for luminal A, 12.5% for basal-like, 12.5% for Her2-overexpressing and 12.5% for unclassified subtype). Patients were managed either by only surgery (9.4%) or by surgery and adjuvant therapy (64.6%). Of this latter group 37.6% had surgery and chemotherapy or targeted therapy (trastuzumab); 26.5% had surgery, chemotherapy, targeted therapy and radiotherapy; 20.5% had surgery, chemotherapy, hormone therapy and radiotherapy; 15.4% had surgery, chemotherapy and hormone therapy.

**Figure 1:**
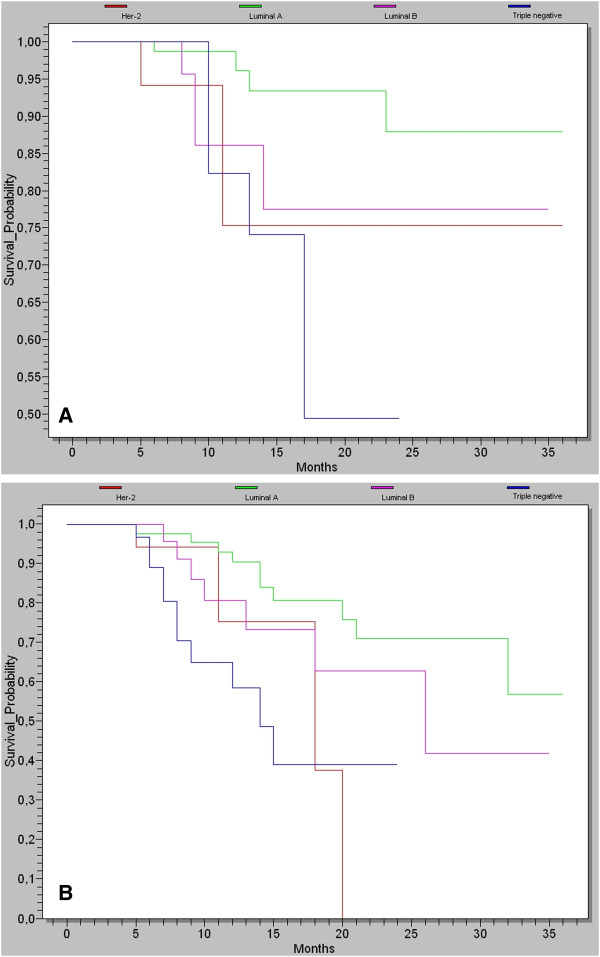
**A: Kaplan–Meier curve illustrating the disease-free survival at 3 years of follow-up.B**: Kaplan–Meier curve illustrating the overall survival at 3 years of follow-up.

In this study, we observed that twenty-two patients (12.2%) died because of cancer-related events during the follow-up. Among these patients, 25% belong to basal-like, 22% belong to unclassified subtype group. 20% to Her2-overexpressing group, 17% to luminal B and only 5% in luminal A. The estimated DFS rate and OS were determined, during 3 years, since the end of the year, in 181 patients recruited in the Medical Oncology Unit. Since basal-like and unclassified tumors were fewer, we gathered them as triple negative breast cancer group. The Kaplan-Meier curves based on the subclasses from Figure1 shows a highly significant difference in OS in 3 years between the subtypes (Figure1A, Log-Rank test: p = 0.042). The triple negative subtype was associated with the lowest survival probability (49%); and the luminal A was associated with the best survival probability (88%) compared to those of the other subtypes (77% for luminal B, 75% for Her2-overexpressing). These subtypes also differed significantly in DFS at 3 years (Figure1B, Log-Rank test: p = 0.002): luminal A (59%), luminal B (41%), triple negative (39%) and Her2-overexpressing (38%).

Multivariate analysis demonstrated that tumor subtypes were associated with both DFS and OS in the group of patients as a whole. Luminal B, Her2-overexpressing and triple negative subtypes were associated with increased relapse compared with luminal A (respectively, HR = 2.38, 95% IC 0.90-6.29; HR = 4.41, 95% C1, 1.35-14.37, HR =4.63, 95% IC 1.93-11.08, p *=0,001*). Similar relationships between OS and different groups were noted: luminal B, Her2-overexpressing and triple negative subtypes had a higher risk than luminal A (respectively, HR =3.24, HR = 5.30, HR =5.19, p = 0.04).

## Discussion

It is well known that breast cancer is a heterogeneous disease characterized by encompassing a wide range of clinical behaviors. Recently, gene expression studies, using microarray technology, confirmed that the heterogeneity of clinical response could be correlated with different molecular profiles of breast cancers [2].

These studies suggested that molecular profiling may be useful in identifying heterogeneity of clinical outcome in breast cancers, which could help clinicians to individualize and improve therapy for their patients.

Although the molecular subtypes of breast cancer were originally identified by gene expression analysis using DNA microarrays, immunohistochemical markers have been used as surrogates in subtyping breast cancer. Based on recent updated IHC subtype definition by Carey et al. [5], we estimated the prevalence of breast cancer subtypes in patients from the north east Moroccan region and established the correlations between clinico-pathological characteristics and DFS and OS.

In this study, the patients recruited in our university hospital were younger than in western series; the average age at diagnosis was 46.8 years with 17% of patient below 35 years and 75% below 55 years. By comparison, in the European population, only 2.7% are below 35 years [9].

In terms of clinical staging, only 14.5% patients were diagnosed at stage I, while 33% were at stage II, 35% at stage III and 17.5% at stage IV. On the other hand, after histological analysis the average tumor size was 3.7 cm and 75% of cases measured more than 2 cm. A majority of tumors were stage II or III, hormone receptor – positive and associated with lymph node involvement. Our data showed that, 53% of patients presented positive lymph nodes and 17.5% of cases had distant metastasis on first diagnosis. This could be due to late consultation during the progression of the disease in our region as well as to lack of the Medicare coverage, lack of screening mammography program and women’s awareness trainings particularly in rural area.

The predominant histology type in this study was invasive-ductal cancer (87.4%), similar to most breast cancer studies worldwide.

ER were expressed in 55.7% of our cases, lower than the mean percentage reported in the literature (60% to 70%) [10].

Overexpression of the protein and/or amplification of the HER2 gene have been reported in approximately 20 to 30% of breast cancers, similar to what was found in our patients (29%). Her2+ tumors are associated with either poor prognosis or with response to trastuzumab [11].

Our results showed a distribution of breast cancer subtypes similar to what was reported by other immunohistochemical studies [5]. Breast cancer subtypes with the ER + Her2– phenotype are the most common, like in our series, corresponding to the luminal A subtype [3].

In this study, the prevalence of luminal B and Her2-overexpressing subtypes was 16.4% and 12.6% respectively. The frequency of these subtypes was weaker in Carolina breast cancer patients (15% of luminal B and 6.6% of Her2-overexpressing subtypes) [5].

Triple-negative subtype represented approximately 17% of our series and 78% of them were basal-like tumors. These results were similar to what was found in other studies [5,12]. The basal-like group was defined by immunohistochemistry, as being negative for ER, PR, and Her2 and positive for Ck5/6, CK14 and/or Her1.

As reported in other series [13-15], the basal-like group was associated with clinico-pathological and biological parameters indicative of high tumor aggressiveness and a worse prognosis: large tumor size (4.4 cm), predominance of high histological grade (47%) and distant metastasis (19%).

The percentage of lymph node positivity (55%) in the basal-like subgroup was the lowest compared with other subtypes. It is possible that this tumor subtype be associated with a predominant hematogenous pattern of dissemination rather than lymphatic; the high incidence of visceral and central nervous system metastases for this subtype as it has been reported [16].

The basal cytokeratins (CK5/6 and CK14) and Her1 expression help identify subtypes of cancers and may differentiate a clinically significant subgroup within the triple negative cancers [17,18]. This group had a poor prognosis regardless the expression of ER or PR [19,20].

Clinically, the basal-like subtype is not only associated with poor clinical outcome, genetic predisposition (BRCA1/2 mutation), high prevalence of p53 mutation and lack of specific biological therapy, but it also has different metastatic patterns [21]. Patients from this group may benefit from EGFR (Her1) targeted therapy [22].

In the absence of specific treatment guidelines for this subgroup, patients with basal-like cancers are unlikely to benefit from currently available targeted systemic therapy and are managed with standard treatment. This evolution is characterized by a high rate of local and systemic recurrence and is associated with aggressive behavior and poor prognosis.

Despite the short follow-up (3 years) in the current study, the OS and DFS were significantly different between breast cancer subtypes.

The luminal A subtype had the highest expression of ER and ER-regulated genes and a better clinical outcome compared to other subtypes, in our study the OS was 88% despite of higher median tumor size (3.6 cm). The luminal B subtype showed lower levels of ER and high levels of genes pertaining to the proliferation cluster [3]. Patients with luminal B, in this study, had a shorter DFS (41%) and a shorter OS (77%) than those obtained for luminal A subtype (60% of DFS and 88% of OS). On the other hand, the luminal B subgroup was associated with high risk for both DFS and OS.

The luminal B subtype in our study presents an aggressive phenotype associated with an intermediate prognosis compared with the luminal A subtype. These data are in accordance with several previous studies [23-25].

The Her2-overexpressing subtype is characterized by ER negativity and high levels of expression of genes pertaining to the HER2 amplicon (17q11), including *HER2GRB7GATA4*, high-level of NF-κB activation [2]. A significant proportion of *HER2*-amplified cancers (i.e. those that are ER-positive) are often associated with luminal B tumors. The DFS and OS for this subtype were 38% and 75% respectively. In this study, only 15% of patients, with positive Her2+ were treated with trastuzumab. This may be explained by the lack of this drug in the hospital during the study period.

The luminal A subtype was associated with a good prognosis, whereas basal-like subtype had a poor prognosis. Moreover, the multivariate analysis showed that this group had a higher risk than luminal A.

In this study, we describe the distinguishing features of these breast cancer subtypes and explain how these features relate both to prognosis and to selection of the most appropriate therapy, such endocrine therapies are the most effective treatments for tumors expressing the estrogen receptor (luminal A and luminal B). Therefore, target therapies (like trastuzumab) are used for patients with profile HER2+, and chemotherapy is effective in tumors with high proliferation. Triple-negative subtype does not respond to hormonal therapy (such as tamoxifen or aromatase inhibitors) or target therapies. However, chemotherapy can be used to treat triple-negative subtype and actually others treatments are under investigations. This diversity of molecular subtype of breast cancer shows a large biological heterogeneity, so each group had a specific genotypic profile wish improve approach to therapy and leads towards personalized therapy of breast cancer.

## Conclusion

We have shown that simple IHC-based classification of breast tumors can be helpful. Since the predictive power of IHC criteria appears to be similar to that of gene expression analysis, this information can be used to improve therapeutic decisions, mainly for luminal B, Her2-overexpressing and basal-like subtypes. The luminal A subtype was associated with favorable biological characteristics and a better prognosis than triple negative tumors that were associated with a poor prognosis and unfavorable clinicopathological characteristics.

In addition, findings concerning tumors stages are alarming and highlight the importance of early screening and the urgent need to improve women’s awareness of breast cancer in our region. Our results should be confirmed by large studies to be conducted in other institutions and hospitals including patients coming from different regions of Morocco.

## Competing interests

The authors declare that they have no competing interests.

## Authors’ contributions

All authors analyzed, interpreted and approved the final manuscript.

## Funding

This study received no specific grant from any funding agency in the public, commercial or not-for-profit sectors.
